# 

**Published:** 1895-11

**Authors:** 


ESTABLISHED 1855. SUBSCRIPTION, $2.00.

### ATLANTA MEDICAL AND SURGICAL JOURNAL.

VOLUME XII. NEW SERIES. ATLANTA, GA., NOVEMBER, 1895. No. 9.

